# The influence of induced moods on aging of phonological encoding in spoken word production: an ERP study

**DOI:** 10.3389/fnhum.2024.1330746

**Published:** 2024-02-13

**Authors:** Lexin Jia, Ruiying Zhao, Qingfang Zhang

**Affiliations:** Department of Psychology, Renmin University of China, Beijing, China

**Keywords:** spoken production, phonological encoding, aging, phonological facilitation effect, induced mood

## Abstract

This study investigated the influence of induced mood on the phonological encoding involved in Chinese spoken word production with a picture-word inference task while concurrently recorded electrophysiological signals. In the experiment, young and older participants watched videos for inducing positive, negative, or neutral mood, and then they were instructed to name target picture while ignoring phonologically related or unrelated distractor words. A phonological facilitation effect was observed in young adults but not in older adults, suggesting an age-related decline of phonological encoding. Both groups showed an inhibition effect in negative mood but not in positive mood, suggesting that speakers have different processing styles in different moods. ERP data revealed a phonological effect around the time window of 250–350 ms in both groups. Meanwhile, young adults showed a phonological effect around 350–450 ms in negative mood and positive mood which may reflect self-monitoring in speech production. We suggest that the former effect may reflect phonological encoding while the latter reflects self-monitoring of internal syllables or phonemes. Furthermore, induced moods influence the phonological effect in older and young adults differently. Behavioral and ERP results provide consistent evidence for the aging decline of phonological encoding in spoken word production.

## 1 Introduction

Spoken word production involves several stages of conceptual preparation (0–175 ms), lexical selection (175–275 ms), phonological encoding (275–355 ms), phonetic encoding (355-450 ms), and articulation (450–600 ms) as revealed by two meta-analyses (Indefrey and Levelt, 2004; Indefrey, 2011). Speakers employ information-universal and linguistic-specific cognitive systems to implement communication coordinately. The former pertains to general cognitive abilities such as processing speed, working memory, and inhibition (Baciu et al., 2016; Boudiaf et al., 2018), whereas the latter pertains to linguistic processing, such as semantic, orthographic, or phonological information (Starreveld and La Heij, 1995; Zhu et al., 2015). Yet, while linguistic aspects and lexical access during speaking are well-investigated, little is understood about how the change of general cognitive abilities and the interplay between information-universal and linguistic-specific factors affect spoken word production. It is a well-established finding that healthy aging is accompanied by decreasing in general cognitive abilities (Park and Reuter-Lorenz, 2009), and mood states modulate processing at several cognitive levels (see Mitchell and Phillips, 2007 for a review), including attention (Schmitz et al., 2009), working memory (Martin and Kerns, 2011), and cognitive control (Dreisbach and Goschke, 2004). With the behavioral and electrophysiological measures, this study aims to investigate how induced mood and phonological information influence the aging of spoken word production in young and older speakers.

Studies have shown an age-related decline in language production at the word and sentence levels. Older adults showed longer reaction times, more pauses, and increased errors when naming pictures (Vousden and Maylor, 2006; Shafto and Tyler, 2014). Compared to young adults, older adults present comparable performance in tasks of semantic judgment (Little et al., 2004), semantic priming (Gold et al., 2009), and homophone words meaning generation task (Kavé and Mashal, 2012). Additionally, older adults experience increased tip-of-the-tongue (hereafter TOT) states, in which speakers can extract meaning but fail to produce the word accurately (Burke et al., 1991; Evrard, 2002). Farrell (2012) conducted a study on semantic priming of TOTs occurrence for both young and older speakers, and found comparable semantic priming effect in both groups. This finding suggests that word retrieval failures in TOTs are little associated with word semantics in both age groups. Most studies have found an aging effect on phonological processing in spoken production. For example, Oberle and James (2013) reported that older speakers benefited more from phonological primes than young speakers when solving TOTs, suggesting that phonological activation may be weaker in older adults (but see Abrams et al., 2007 for an age invariant between young and young-old adults).

There are two assumptions to account for the aging of spoken word production. One is *the transmission deficit hypothesis* (hereafter TDH) from the perspective of the linguistic-specific framework (MacKay and Burke, 1990; Burke et al., 1991; Burke and Shafto, 2004). The TDH assumes that the connection between semantics and phonology weakens with age, results in a reduction in the transmission from semantics to phonology, and difficulty in activating phonological representations. Empirical findings provide evidence for the TDH. For instance, studies reported a dissociation between semantic and phonological retrievals during TOTs occurrence (Thornton and Light, 2006; Burke and Shafto, 2008). Vigliocco et al. (1999) observed successful retrieval of lexical-syntactic properties (e.g., countability, grammatical gender, and number) of target words yet inability to retrieve complete phonological segments of target words (see also Schwartz, 2002). Studies showed that semantic primes do not reduce TOT occurrence or improve TOT resolution (Farrell, 2012; Kumar et al., 2019), whereas phonological primes reduce TOTs occurrence (Burke and Shafto, 2004; Farrell and Abrams, 2011; Pureza et al., 2013) and improving TOTs resolution (Oberle and James, 2013; White et al., 2013) significantly.

Alternatively, information-universal theories assume that the declines of general cognitive abilities such as processing speed, working memory, inhibitory, and executive control result in the age-related decline of spoken word production (Hasher et al., 2007; Park and Reuter-Lorenz, 2009; Ebaid et al., 2017). Numerous studies have shown that the declines of non-specific cognitive abilities lead to not only increased latencies in picture naming and semantic processing (Shao et al., 2012; Boudiaf et al., 2018) but also highly dysfluency speech (Mortensen et al., 2006; Engelhardt et al., 2013; Korko and Williams, 2017). Hasher and colleagues proposed the *inhibition deficit hypothesis* (hereafter IDH) to explain the distinction between young and older adults, which assumes that healthy aging disrupts inhibitory mechanisms, rendering the reduced ability to suppress irrelevant or competing information (Hasher and Zacks, 1988; Hasher et al., 2007). Studies of spoken word production provide empirical evidence for the IDH. In a rapid naming task, older speakers produced more errors than young speakers, indicating that older adults cannot suppress irrelevant words in a time-limited situation (Neumann et al., 2018). There is a significant correlation between the Stroop effect and the semantic interference effect in older adults (Crowther and Martin, 2014), implying the role of inhibitory ability in spoken word production.

It is well-known that mood states modulate cognitive processing at various levels, including attention (Schmitz et al., 2009), working memory (Martin and Kerns, 2011), decision making (Hockey et al., 2000), problem solving (Gasper, 2003), cognitive control (Dreisbach and Goschke, 2004), and phonological processing in spoken production (Hinojosa et al., 2017). These findings suggest that mood state can influence cognitive processes as an information-universal factor, and several hypotheses attempt to explain the mechanisms of mood's influences on cognition (Martin and Clore, 2013). The *affect-as-information theory* assumes that different moods influence the processing styles used (Bodenhausen et al., 1994; Clore and Huntsinger, 2007). Specifically, individuals experiencing positive moods prefer to use a more global and flexible heuristic processing style, but an analytical and effortful processing style when experiencing negative moods. In contrast, the *attention theory* proposes that moods modulate cognitive processing by regulating attention (Derryberry and Tucker, 1994; Fredrickson, 2001; Förster et al., 2006). Positive moods are thought to broaden the scope of attention, while negative moods restrict it (Derryberry and Tucker, 1994; Fredrickson, 2001; Förster et al., 2006), potentially affecting processing efficiency. Alternatively, the *capacity limitation theory* argues that the cognitive resources available for task-related information processing are distinct. Processing neutral mood information requires fewer cognitive resources, while processing information with positive or negative mood requires more cognitive resources (Ellis and Ashbrook, 1988; Schmeichel, 2007).

There are considerable evidences confirming that several processes involved in language comprehension are modulated by mood state. Behavioral studies report that comprehending words, sentences, or discourses is facilitated when the emotional meaning of the stimuli is congruent with one's mood (e.g., Ferraro et al., 2003; Egidi and Caramazza, 2014). Furthermore, studies have shown that compared to negative mood, positive mood improves semantic integration (e.g., Chwilla et al., 2011; Pinheiro et al., 2013), the detection of semantic reversal anomalies (Vissers et al., 2013), and the anticipation of referents (Van Berkum et al., 2013). Additionally, neuroimaging investigations have identified that different cognitive mechanisms underlying positive and negative mood processing, evidenced by distinct activation of brain regions (Davidson, 2003; Matsunaga et al., 2009; Vogt, 2009; Kohn et al., 2014; Tsujimoto et al., 2022). Specifically, positive mood processing is associates with increased activation of the pregenual part of the anterior cingulate cortex, prefrontal cortex (Matsunaga et al., 2009; Vogt, 2009; Tsujimoto et al., 2022), and left prefrontal cortex (Davidson, 2003), while negative mood processing associated with activations in the subgenual anterior cingulate, the ventromedial frontal cortex (Vogt, 2009; Kohn et al., 2014) and right prefrontal cortex (Davidson, 2003).

However, little is known about the impact of mood state on language production. Three separate studies, including one behavioral (White et al., 2016) and two electrophysiological studies (Hinojosa et al., 2010, 2017), suggest that phonological processing involved in word production is particularly sensitive to various mood states. Using a picture-word interference task, White et al. (2016) found that taboo words have the greatest phonological promotion effect when compared to positive, neutral, and negative words, revealing that the valence of emotional words modulates the phonological promotion effect. With electrophysiological measurements, Hinojosa et al. (2010) observed that monitoring grapheme of picture names with positive and negative emotions yielded slower reaction times and larger positive shift waveforms at around 400 ms after picture onset compared to the those with neutral picture names. Hinojosa et al. (2017) first instructed participants to watch a short film clip to induce negative, positive, or neutral moods and then asked participants to perform a picture naming task. They reported that ~290 ms after pictures onset, watching short film clips that induce negative mood elicits larger negative shift waveforms than neutral mood conditions. However, there was no significant difference between positive and neutral moods. The time intervals around 290 or 400 ms primarily tap into phonological processing in spoken word production (Indefrey and Levelt, 2004; Indefrey, 2011), thus, Hinojosa and colleagues propose that phonological processing is particularly sensitive to mood or affective state.

Meanwhile, a growing body of empirical research has shown that mood processing styles differ between young and older adults. Healthy aging is associated with decreased neural response (Kisley et al., 2007) and decreased attention (Mather and Carstensen, 2003; Murphy and Isaacowitz, 2008) toward negative mood. Moreover, older adults report experiencing more positive mood and higher mood stability than young adults (Carstensen et al., 2000, 2011). This may be related to the age-related positivity effect, in which automatic cognitive processing is inclined to positive information such as attention and memory (Charles et al., 2003; Mather and Carstensen, 2003). Specifically, compared to young adults, older adults report a greater preference for positive information (Murphy and Isaacowitz, 2008) or a stronger preference to avoid/reduce negative information (Kisley et al., 2007). For instance, older adults recall a higher proportion of positive stimuli than negative stimuli, whereas young adults exhibit the opposite pattern (Charles et al., 2003; Langeslag and van Strien, 2009).

There are several theoretical approaches to explain the underlying mechanisms of distinct mood processing between young and older adults. A widely accepted view is the *socioemotional selectivity theory* (henceforth SST), which assumes that older adults select positive social experiences and emotional satisfaction because they are aware of the transience of future time (Charles et al., 2003). While young adults with a more expansive future time, tend to seek more present mood experiences to learn new information and develop new relationships. Alternatively, the *connected theoretical view* assumes that, due to prolonged training and experience over time, older adults have more effective emotional regulation and use fewer cognitive resources than young adults (Scheibe and Blanchard-Fields, 2009). The model of *conceptualizing emotional development* claims that the ability to integrate emotional and cognitive information increases with age, approaching peaks in middle age. After that, this capacity declines slightly in old age but continues to remain high overall (Labouvie-Vief et al., 1989). Based on this assumption, older adults are able to maintain their problem-solving capacity under emotional situations, while young adults show a lower performance when compared with older adults (Blanchard-Fields et al., 1995; Labouvie-Vief, 2003).

However, cognitive aging does not always imply a decline in cognitive performance, and older adults can perform well as young adults by adopting processing strategies to compensate for their disadvantages (e.g., Wingfield and Grossman, 2006; Meinzer et al., 2009). Studies of cognitive aging using functional neuroimaging have shown that older adults have decreased activation in the occipitotemporal region and increased frontal activity compared to young adults (Daselaar et al., 2003). Older adults exhibit bilateral prefrontal cortex activation, whereas young adults exhibit lateral prefrontal cortex activation. This suggests a hemispheric asymmetry reduction in older adults (Cabeza, 2002), indicating that older adults may have different activation patterns at the brain level.

Although general cognitive abilities such as processing speed, working memory, and inhibition, decline as part of the healthy aging process (Park and Schwarz, 2000; Salthouse et al., 2003), older adults probably adopt different strategies for completing spoken production in varying moods in which involves factors of information-universal (e.g., mood) as well as linguistic-specific ones (e.g., semantics, phonology). So far, little is known about how mood modulates the aging effect in spoken word production. Using a picture naming task, Huang et al. (2017) found that speaking naming latencies are longer in negative emotional condition than positive or neutral emotional ones, suggesting an inhibition effect for negative mood. Furthermore, this inhibition effect is greater in older adults than in young adults. Blackett et al. (2017) found that naming latencies for positive and negative images were longer than those for neutral images, and older adults presented greater discrepancy between emotional (positive or negative) and neutral images than young adults. These findings indicate that moods influence cognitive-aging phenomena.

Electroencephalography (EEG) is a well-established approach employed widely in language research providing high time-resolution measurements for stages involved in language processing (Bürki and Laganaro, 2014; Valente and Laganaro, 2015; Zu Wolfsthurn et al., 2021). Our study aims to investigate the influence of mood on the aging of spoken word production in picture naming utilizing the EEG approach. We compare young and older adults using a picture-word interference (henceforth PWI) task. In the PWI experiment, participants were instructed to watch videos for inducing different moods (positive, negative, and neutral), and then complete the PWI task, in which phonological relatedness between distractors and target names (phonologically related and unrelated) was manipulated. Numerous studies have demonstrated that phonologically related distractors shorten picture naming latencies when compared with unrelated distractors (Starreveld and La Heij, 1995). Studies in Chinese spoken production have shown that phonologically related distractors tap into phonological encoding (Zhu et al., 2015; Zhang and Damian, 2019), which occurs in a time window of around 250–450 ms in spoken word production (Indefrey and Levelt, 2004; Indefrey, 2011).

We have three specific objectives: (1) to investigate the impact of induced mood on the phonological facilitation effect in spoken word production, (2) to identify varying patterns of the interplay between induced mood and phonological relatedness in young and older adults, and (3) to determine the temporal courses of these effects in two age groups. At the behavioral level, studies found that negative mood inhibits spoken production (Huang et al., 2017) and specifically inhibits phonological encoding stage (Hinojosa et al., 2017), that is, negative mood would lengthen speaking latencies. We thus predict a reduced phonological facilitation effect in negative moods compared to neutral and positive moods. Furthermore, as above-mentioned, young and older adults have distinct styles in processing negative and positive moods. Meanwhile, young and older adults show different phonological facilitation effect in spoken word production (i.e., James and Burke, 2000; White and Abrams, 2002). We thus expect a triple interaction among age, induced mood, and phonological relatedness. For the EEG measurement, we predict a triple interaction among age, induced mood and phonological relatedness (see also Hinojosa et al., 2010, 2017), and the interaction between induced mood and phonological relatedness would be different in young and older adults, in the time window of 250–450 ms.

## 2 Methods

### 2.1 Participants

Twenty-four young adults (16 females and eight males, 19–20 years; *M* = 20.08, *SD* = 1.47) from Renmin University of China and 24 older adults (11 females and 13 males, 61–69 years; *M* = 65.87, *SD* = 3.57) from local communities around university's campus participated in the experiment. The sample size was estimated using G^*^Power v.3.1 (Faul et al., 2009), and results indicated that 18 participants per group were required to achieve an 80% power for detecting an effect size of 0.3 at a significant level of α = 0.05 for an analysis of variance with repeated measures of two factors. All participants were native Mandarin Chinese speakers who were neurologically healthy with normal or corrected-to-normal vision, no hearing loss, and no language impairments. All older adults scored 26 or higher (M = 27.65, SD = 1.18) on the Montreal Cognitive Assessment (henceforth MoCA), reflecting that they were in healthy aging status (Nasreddine et al., 2005). The age groups matched in educational years, *t*_(46)_ = 0.491, *p* = 0.68. All participants gave their informed consent before the experiment and were paid for their participation. The study was approved by the Research Ethics Committee of the Department of Psychology, Renmin University of China.

### 2.2 Stimuli

We selected 70 target black-and-white-line pictures with disyllabic names from the Chinese standardized picture database (Zhang and Yang, 2003), of which 60 were used in experimental trials and 10 were used in practice trials. Each picture was paired with a phonologically related distractor prime that shared an atonal syllable (same phonemes but different tone) with the first syllable of the target name. For instance, the target name 信封 (/xin4.feng1/, envelope) was matched with a phonologically related distractor prime 新闻 (/xin1.wen2/, news). The same pictures and distractors were re-paired to create 60 unrelated distractor-target pairs, in which semantic or orthographic overlap between distractors and targets was avoided. All pictures and distractor words are presented in Supplementary material.

**Figure 1:**
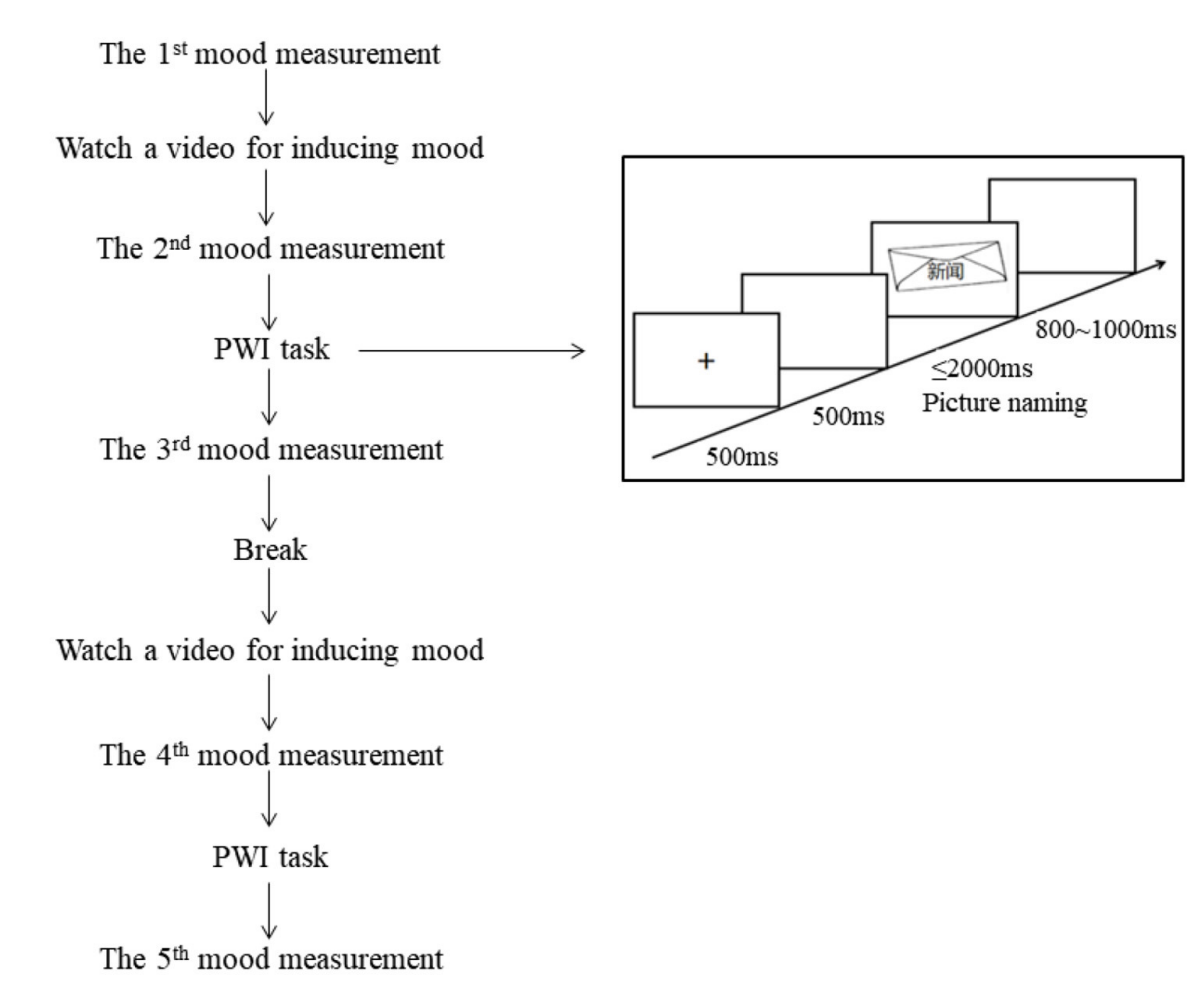
A schema of each block in the experiment.

We selected six videos from the Native Chinese Affective Video System (Xu et al., 2010) for inducing different moods, and two of them were used for inducing moods of the positive, negative, and neutral states, respectively (Table 1). The arousal intensity of this database was assessed using a self-reported 5-point scale: “When I was watching the video…,” with 0 representing “I felt no emotions at all” to 4 representing “I felt very intense emotions.” The emotional valence and discreteness were assessed by the Positive and Negative Affect Schedule and the Differential Emotions Scale (see Schaefer et al., 2010 for details) using a 5-point scale. After determining the dominant emotional valence of each video based on the assessing values, the discreteness was computed separately for each video by the percentage of participants (50 in total) reporting the dominant emotional valence (i.e., sad, fear, anger, happy, disgust, or neutral). After that, they selected five videos for each emotional valence, and ranked them from low (1) to high (5) scores along the dimension of arousal intensity and discreteness, separately. Xu et al. (2010) presented the ranked orders in the database, and did not report the specific scores for discreteness and arousal intensity. Figure 1 shows the ranked orders of each video for the discreteness and arousal intensity, separately. Xu et al. (2010) demonstrated that these videos can effectively elicit the intended emotional responses in participants.

**Table 1:** The properties of each video.

**Mood**	**Video**	**Discreteness**	**Intensity**
Positive	≪Eat Hot Tofu Slowly≫	1	2
	≪A Big Potato≫	2	4
Neutral	≪IDE Interface Repair≫	1	1
	≪Repair Computer≫	2	4
Negative	≪Roots and Branches≫	2	1
	≪Warm Spring≫	4	3

### 2.3 Design

We adopted a 2 (age: young vs. older) × 2 (phonological relatedness: related vs. unrelated) × 3 (induced mood: positive, negative, and neutral) mixed design. The age was a between-participants and within-items factor, phonological relatedness and induced mood were within-participants and within-items factors. The induced mood was blocked, and the order of mood blocks was counterbalanced among participants according to a Latin square design. In order to avoid the mutual influence among moods, participants performed three blocks separately with a week interval. Each block consists of two sets, and a participant saw each picture twice (one in related and the other in unrelated) for a total of 120 trials in each set. This set was repeated two times, thus each block with one mood state consisted of 240 trials. The entire experiment consisted of 720 trials. The sequence of trial presentations was pseudo-randomized to avoid the continuous presentation of the same picture or the continuous presentation of the same interfering word. Before each set, participants watched one video for inducing a mood state, and the order of two videos with the same mood was counterbalanced among participants. There was a break between two sets with a maximum period of 5 min.

A 9-point valence rating scale was used to assess whether the target mood was successfully induced (Zhang et al., 2014) during the experiment, with one indicating highly negative, five indicating neutral, and nine indicating highly positive (Table 2). The initial emotional state was measured before each block, while the second and fourth assessments were conducted to measure the emotional state elicited immediately after watching a mood video. The third and fifth assessments were conducted to measure the maintained emotional state after completing the picture-word interference task. Please refer to Figure 1 for a detailed schema of each block (see Figure 1 for a detailed schema of each block).

**Table 2:** The mean scores of rating mood states during the experiment.

	**Young**	**Older**
**Mood states**	***M (SD)***	***M (SD)***
Baseline	5.85 (1.08)	6.30 (1.26)
Positive Evoked I	6.80 (1.19)	7.30 (1.45)
Positive Evoked II	6.30 (1.26)	7.00 (1.29)
Baseline	6.00 (1.21)	6.05 (1.35)
Negative Evoked I	4.85 (1.46)	5.25 (1.77)
Negative Evoked II	4.25 (1.44)	4.75 (2.24)
Baseline	5.75 (1.01)	5.45 (1.23)
Neutral Evoked I	5.05 (1.43)	5.65 (1.09)
Neutral Evoked II	5.35 (1.08)	6.10 (1.08)

### 2.4 Apparatus

The experiment was administered using E-Prime Professional Software 2.0. Stimuli were presented on a high-resolution monitor of 1,024 × 768. Naming latencies were measured from target onset using a voice key, connected to the computer via a PST Serial Response Box. Neuroscan 4.3 software was used to record EEG signals.

### 2.5 Procedure

Participants were seated individually in front of a computer screen in a quiet room. Before the experiment, participants were instructed to familiarize themselves with the target pictures by viewing each target for 3,000 ms with the correct picture name presented below each picture. After the learning phase, participants received a picture naming test without concurrently presenting names. When all pictures were correctly named, the experimental block was administered, comprising 20 practice trials and 240 trials per block.

Each mood block involved the following sequence: a 9-point scale was presented, and participants were asked to evaluate their current mood state. Then, they watched a video for inducing the target mood, and then evaluated the mood state again. After that, participants completed one set including 10 practice trials and 120 target trials, and evaluated their mood state on a 9-point scale. After the break, participants watched the second video that induced the same mood as the first one, completed the second set, and evaluate the mood state finally.

Each trial involved the following sequence: a fixation point (+) was presented at the center of the screen for 500 ms, followed by a blank screen for 500 ms. Subsequently, a target picture was presented on which a distractor was superposed at the center of the target picture. Participants were instructed to ignore the distractors and name the pictures as accurately and quickly as possible. The picture disappeared once the participant initiated a voice response, or after a time-out of 2,000 ms. A random inter-trial interval ranging from 800 to 1,000 ms concluded in each trial. Following each response, the experimenter judged and recorded whether the response was correct or not. Naming latencies were measured from the onset of the picture to the onset of articulation.

### 2.6 EEG recording and analysis

The experiment used the ESI-64 electrodes recording system produced by NeuroScan Company in the United States and Neuroscan 4.3 software. During the recording of electrophysiological signals, the left mastoid was used as the reference. Both vertical electrooculogram (VEOG) and horizontal EOG (HEOG) were bipolar recorded. The VEOG electrodes were placed in the middle of the upper and lower orbits of the left eye, and the HEOG electrodes were placed 1 cm on both sides of the left and right eye corners. The scalp resistance at each electrode remained below 5 kΩ. During the experiment, electrophysiological signals were amplified with a bandpass filter of 0.05 and 100 Hz and digitized continuously at a sampling rate of 500 Hz.

The data were then processed offline using the EEGLAB software package (Delorme and Makeig, 2004). All single-trial waveforms were screened for eye movements, electrode drifting, amplifier blocking, and artifacts. EEG data were re-referenced offline to the average of the two mastoids (M1 and M2; see also Chwilla et al., 2011; Hinojosa et al., 2017; Cai et al., 2023; Zhang et al., 2023). Epoch was from 200 ms before the presentation of the picture to 800 ms after the presentation of the picture, with a baseline correction from 200 to 0 ms preceding the target pictures onset. Offline filtering used a 30 Hz (24 dB/oct) low-pass filter and a 0.1 Hz high-pass filter. Epochs containing artifact signals below/above 100 μV were rejected. Trials in which speakers produced incorrect responses and those with naming latencies faster than 500 ms or longer than 2,000 ms were excluded from the EEG analysis.

To determine the time windows where the mean amplitude of each electrode varies significantly differences in both cases, we performed a traditional waveform analysis. Nice ROIs were selected based on the sagittal and coronal axes: left-anterior (FC3), left-central (C3), left-posterior (P3), mid-anterior (FCZ), mid-central (CZ), mid-posterior (PZ), right-anterior (FC4), right-central (C4), and right-posterior (P4).

Mean amplitudes were calculated for each participant and condition in the two-time windows from 250 to 450 ms, with a step increase of 100 ms. For each time window, mean amplitudes were entered into a repeated measures ANOVA with the factors of age, phonological relatedness, and regions of interest (henceforth ROI). The Greenhouse-Geisser correction was applied to all repeated measures with more than one degree of freedom. To control for type I errors in multiple comparisons under different time windows, we used the false discovery rate (FDR) method (Yekutieli and Benjamini, 1999), as implemented in R software using the *fdrtool* package. We report the corrected *p*-values of the simple effect in each time window.

## 3 Results

Eight participants including four young and four older adults were excluded from the analysis due to large electrode drift and excessive artifacts. To assess whether the mood state was successfully induced, we conducted paired *t*-tests to compare the mood scores between the baseline (1st assessment) and the induced mood state (2nd or 4th assessment) in each mood block, for young and older groups separately. For young adults, the difference in positive mood was significant between 1st vs. 2nd, *t*_(19)_ = −4.25, *p* < 0.001, and marginally significant between 1st vs. 4th, *t*_(19)_ = −1.93, *p* = 0.06; the differences in negative mood were significant between 1st vs. 2nd, *t*_(19)_ = 3.04, *p* < 0.01, and between 1st vs. 4th, *t*_(19)_ = 4.65, *p* < 0.001; the differences in neutral mood were not significant between 1st vs. 2nd, *t*_(19)_ = 1.51, *p* > 0.1, and between 1st vs. 4th, *t*_(19)_ = 1.32, *p* > 0.1. For older adults, the difference in positive mood was significant between 1st vs. 2nd, *t*_(19)_ = −2.56, *p* < 0.05, and marginally significant between 1st vs. 4th, *t*_(19)_ = −1.85, *p* = 0.08; the difference in negative mood was marginally significant between 1st vs. 2nd, *t*_(19)_ = 1.89, *p* = 0.07, and significant between 1st vs. 4th, *t*_(19)_ = 2.33, *p* < 0.05; the differences in neutral mood were not significant between 1st vs. 2nd, *t*_(19)_ = −1.00, *p* > 0.1, and between 1st vs. 4th, *t*_(19)_ = −1.55, *p* > 0.1.

To examine whether or not participants maintained the emotional state throughout the block, we performed paired *t*-tests to compare the emotional scores between baseline (the first assessment) and induced emotional states (the third or the fifth assessment) in each emotional block, for young and older groups separately. For young adults, the differences in positive mood were marginally significant between 1st vs. 3rd, *t*_(19)_ = −1.83, *p* = 0.08, and between 1st vs. 5th, *t*_(19)_ = −1.79, *p* = 0.08; the differences in negative mood were significant between 1st vs. 3rd, *t*_(19)_ = 2.41, *p* < 0.05, and between 1st vs. 5th, *t*_(19)_ = 2.88, *p* < 0.01; the differences in neutral mood were not significant between 1st vs. 3rd, *t*_(19)_ = 1.43, *p* > 0.1, and between 1st vs. 5th, *t*_(19)_ = 1.55, *p* > 0.1. For older adults, the difference in positive mood was marginally significant between 1st vs. 3rd, *t*_(19)_ = −1.78, *p* = 0.09, but insignificant between 1st vs. 5th, *t*_(19)_ = −1.36, *p* = 0.18; the differences in negative mood were marginally significant between 1st vs. 3rd, *t*_(19)_ = 1.81, *p* = 0.08, and between 1st vs. 5th, *t*_(19)_ = 1.89, *p* = 0.07; the differences in neutral mood were not significant between 1st vs. 3rd, *t*_(19)_ = −1.67, *p* > 0.1, and between 1st vs. 5th, *t*_(19)_ < 1, *p* > 0.1.

### 3.1 Behavioral results

Incorrect response trials (1.6%), naming latencies shorter than 500 ms or longer than 2,000 ms (3.9%), and naming latencies deviating more than three standard deviations (1.2%) were excluded. Table 3 presents the mean naming latencies and standard deviations of correct trials by age, induced moods, and phonological relatedness.

**Table 3:** Mean reaction times (RT in ms) and error rate (%) for phonologically related and unrelated conditions under positive, neutral, and negative moods in young and older adults.

**Induced mood**	**Young**				**Older**			
	**Related**		**Unrelated**		**Related**		**Unrelated**	
	**RT (SD)**	**ER (%)**	**RT (*SD*)**	**ER (%)**	**RT (*SD*)**	**ER (%)**	**RT (SD)**	**ER (%)**
Positive	802 (166)	1.40	817 (170)	1.70	837 (188)	1.70	840 (183)	1.60
Neutral	796 (178)	1.50	819 (180)	1.50	836 (185)	1.40	839 (173)	1.10
Negative	812 (178)	3.10	837 (179)	1.00	849 (195)	1.60	857 (183)	2.10

We used the *lmer* program of the *lmeTest* package to estimate fixed effects and parameters of the linear mixed effect model (Bates, 2005; Baayen et al., 2008) with R (R Development Core Team, 2009). Latencies were analyzed using a linear mixed-effects model that included fixed factors of age, induced mood, phonological relatedness, and their interactions with participants blocks and items as random intercepts and slope adjustments for all fixed effects. The model adopts a restricted maximum likelihood estimation method, which uses the optimal parameter estimation of the best matching model for observation data. The most adjustment model that significantly improved the variance estimation over previous models was the best-fitting model.

The best-fitting model was determined by the following steps (Bates et al., 2015). We first specify a model (i.e., null model) that included only random factors (participants, block, and items); we then enrich the null model by adding fixed factors including age, induced mood, phonological relatedness, the interaction between age and induced mood, the interaction between age and phonological relatedness, the interaction between phonological relatedness and induced mood, and the triple interaction among age, induced mood, and phonological relatedness. Third, we compared the newly established model to a previous model using the chi-square test. If adding a fixed factor, an interaction between two variables, or a triple interaction among three variables to an existing model did not significantly improve the variance estimation, then the current model is the best fitting one.

**Figure 2:**
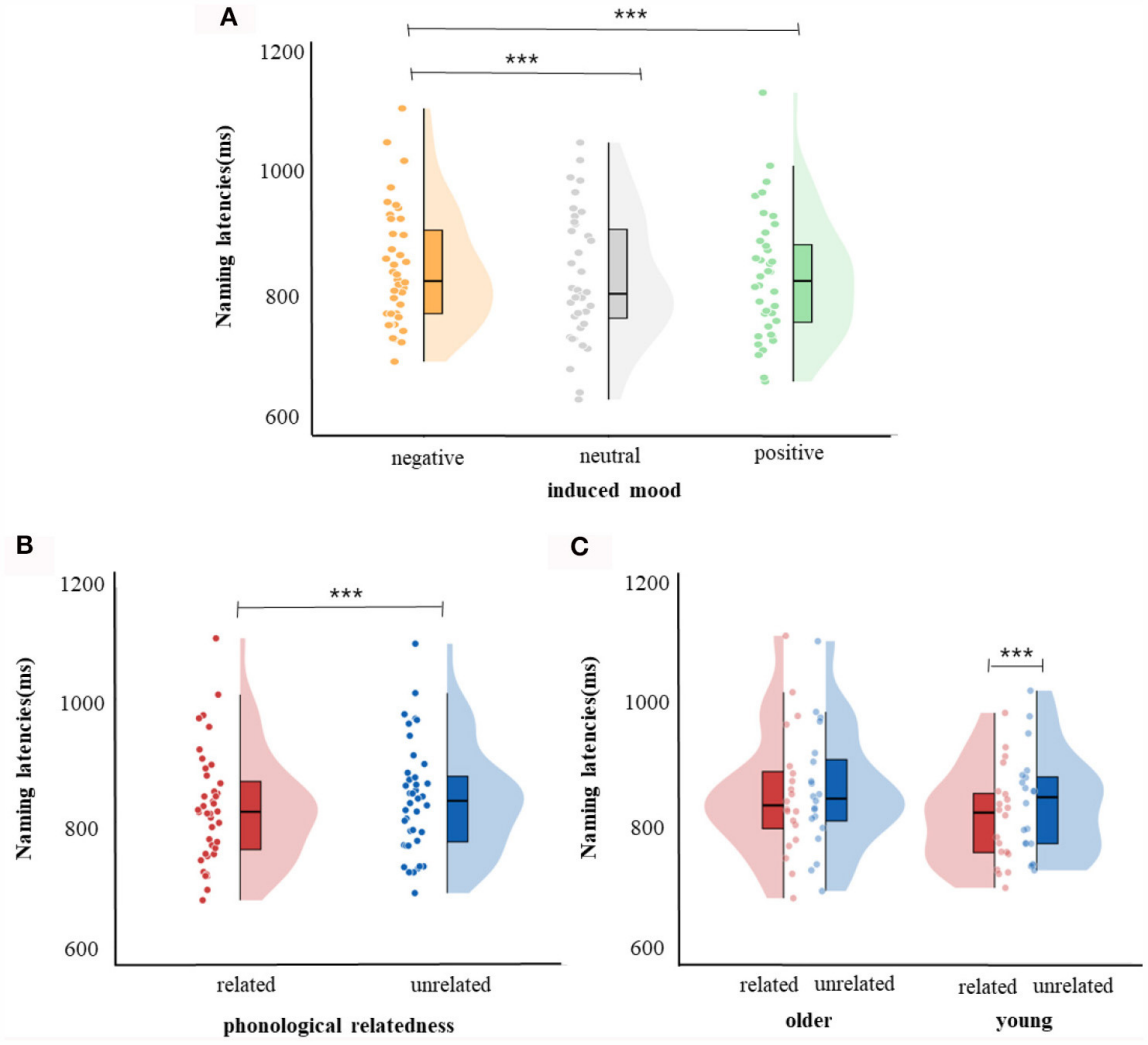
Naming latencies. **(A)** Violin plots for negative, neutral, and positive mood conditions. **(B)** Violin plots for phonologically related conditions and phonologically unrelated conditions. **(C)** Violin plots for phonologically related conditions and phonologically unrelated conditions in young and older group. The violin plot outline shows the density of data points for different naming latencies, the boxplot shows the interquartile range with the 95% confidence interval represented by the thin vertical black line. Full dots represent individual data points. Black bars represent median. ***p < 0.001.

For response latencies, the best-fitting model was RT ~ phonological relatedness + induced mood + age: phonological relatedness + (1| participant) + (1| block) + (1| items).^1^ Results showed (Table 4) that the effect of induced mood was significant, latencies were significantly shorter in the positive than in the negative (14.75 ms), *estimate* = −15.58, *SE* = 3.12, *t* = −4.98, *p* < 0.001; and latencies were significantly shorter in the neutral than the negative (16.25 ms), *estimate* = −16.86, *SE* = 3.13, *t* = −5.39, *p* < 0.001 (Figure 2A). The effect of phonological relatedness was significant, *estimate* = 21.98, *SE* = 3.58, *t* = 6.137, *p* < 0.001, with naming latencies faster in the phonologically related than the unrelated condition (12.83 ms; Figure 2B). The interaction between age and phonological relatedness was significant, *estimate* = −0.83, *SE* = 0.32, *t* = −2.58, *p* < 0.01 (Figure 2C). Further simple effect analysis with *emmeans* package showed that for young adults, naming latencies in the phonologically related condition was significantly shorter than the unrelated condition (21 ms), *estimate* = −21.98, *SE* = 3.58, *t* = −6.14, *p* < 0.001(*p* < 0.001 after *Bonferroni* correction), for older adults, naming latencies in the phonologically related condition was not significantly shorter than the unrelated condition (4.66 ms), *estimate* = −6.04, *SE* = 3.64, *t* = −1.66, *p* = 0.09 (*p* = 0.57 after *Bonferroni* correction).

**Table 4:** Summary of the best-fitting model for naming latencies.

**Predictors**	**β**	***SE***	***df***	***t***	***p***
Intercept	815.56	23.09	36.69	35.32	< 0.001
Induced mood (positive—negative)	−15.59	3.13	133,315.26	−4.99	< 0.001
Induced mood (neutral—negative)	−16.86	3.13	133,315.38	−5.39	< 0.001
Phonological relatedness	21.98	3.58	133,315.13	6.14	< 0.001
Age: phonological relatedness	−15.938	5.11	133,315.24	−3.122	< 0.01

### 3.2 ERP results

Trials were excluded based on the procedure of analyzing EEG signals. For young adults in the phonologically related condition, 13.33, 16.25, and 13.83% of trials were excluded in positive, negative, and neutral moods, respectively, while in the phonologically unrelated condition, 11.42, 14.67, and 14.92% of trials were excluded in positive, negative, and neutral moods, respectively. For older adults in the phonologically related condition, 11.08, 15.25, and 14.08% of trials were excluded in positive, negative, and neutral moods, respectively, while in the phonologically unrelated condition, 11.00, 15.83, and 11.42% of trials were excluded in positive, negative, and neutral moods, respectively. The remaining trials were applied in the below waveforms analysis.

**Figure 3:**
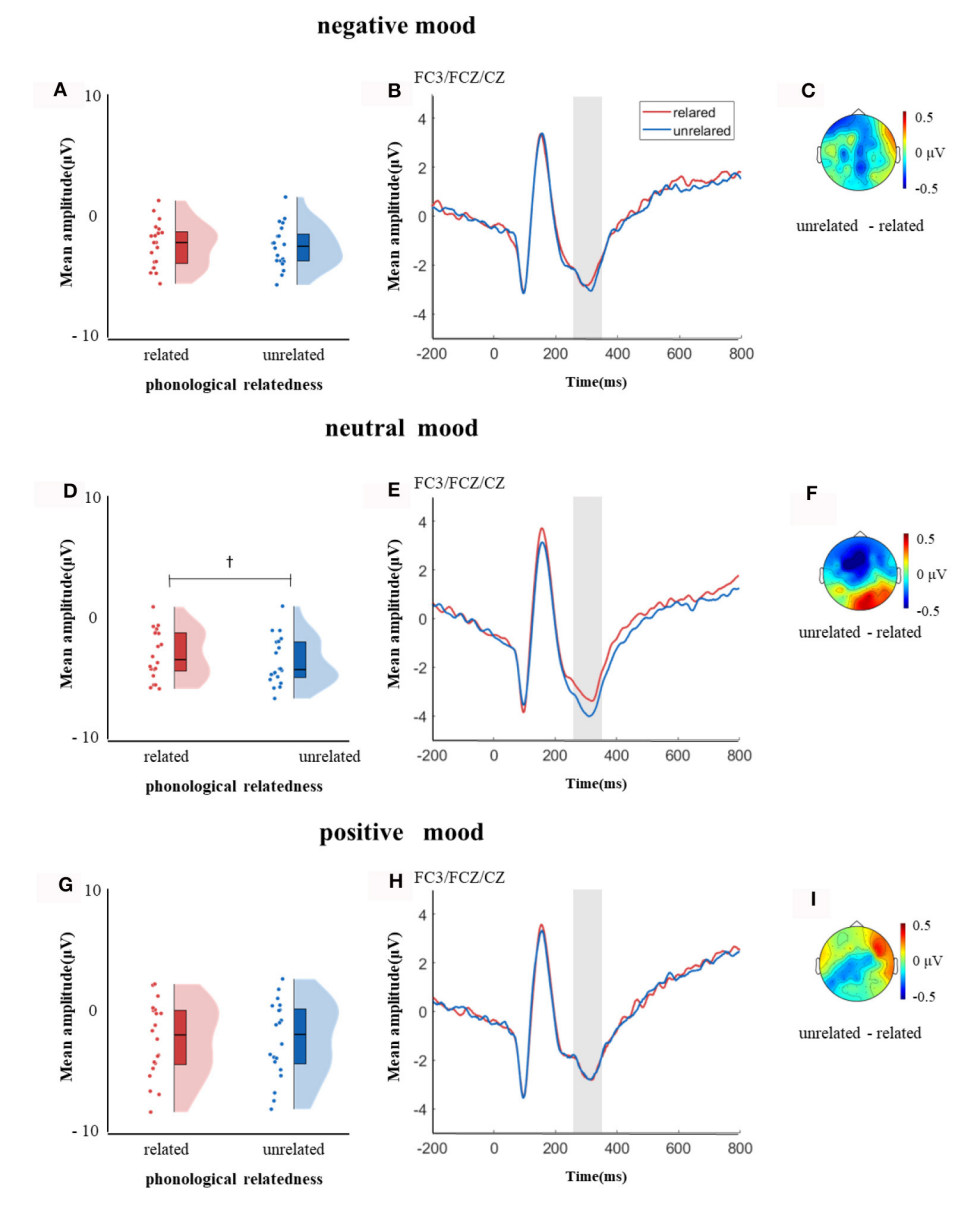
The mean amplitude in the time window of 250–350 ms (the shadowed interval). **(A, D, G)** Violin plots of phonologically related and unrelated in different induced moods, **(B, E, H)** the grand average waveforms for phonologically related and unrelated in different induced moods, and **(C, F, I)** the map distributions for the significant differences between phonologically related and unrelated in different induced moods, with negative, neutral and positive mood all at left-posterior, mid-posterior and mid-central region. ^†^0.05 < *p* < 0.1.

**Figure 4:**
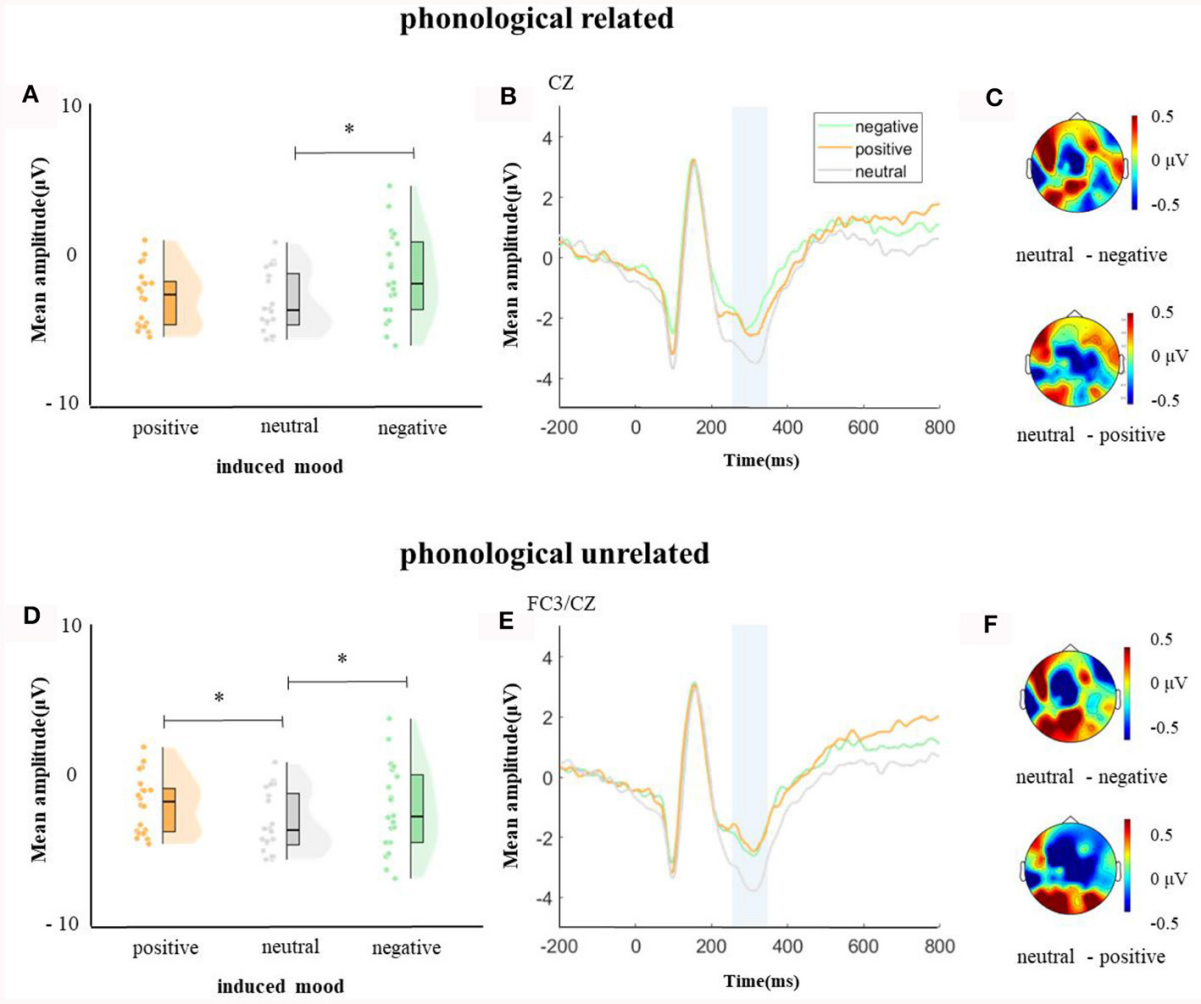
The mean amplitude in the time window of 250–350 ms (the shadowed interval). **(A, D)** Violin plots of different induced moods in phonologically related and unrelated conditions, **(B, E)** the grand average waveforms for different induced moods in phonologically related and unrelated conditions, and **(C, F)** the map distributions for different induced moods in phonologically related and unrelated conditions, with phonologically related condition at mid-central, and phonologically unrelated condition at left-posterior and mid-central region. *p < 0.05.

In the time window of 250–350 ms after pictures onset, the effect of age was significant, *F*_(1, 38)_ = 6.37, *p* = 0.04, $\eta_{p}^{2}$ = 0.14, older adults elicited a smaller negative waveform than young adults; and the interaction between age and ROI was significant, *F*_(8, 304)_ = 4.72, *p* < 0.001, $\eta_{p}^{2}$ = 0.11, simple effect analysis (after *FDR* correction) showed that older adults elicited a smaller negative waveform than young at left-anterior (*p* = 0.004), left-central (*p* = 0.034), left-posterior (*p* = 0.019), mid-anterior (*p* = 0.009), and right-anterior (*p* = 0.02). The effect of phonological relatedness was significant, *F*_(1, 38)_ = 4.75, *p* = 0.04, $\eta_{p}^{2}$ = 0.11, and phonologically related condition elicited a smaller negative waveform than phonologically unrelated condition. The triple interaction among phonological relatedness, induced mood, and ROI was significant, *F*_(16, 608)_ = 2.40, *p* = 0.002, $\eta_{p}^{2}$ = 0.06, and further simple effect analysis (after *FDR* correction) showed that in the neutral mood, the phonologically related condition elicited a smaller negative waveform than the unrelated condition at ROIs of left-anterior (*p* = 0.009), mid-anterior (*p* = 0.049) and mid-central (*p* = 0.076). There was no significant difference between phonologically related and unrelated in negative or positive mood at all ROIs (all *ps* > 0.1; Figure 3). In the phonologically related condition, the negative mood elicited a smaller negative waveform than the neutral at ROIs of mid-central (*p* = 0.03), while in the phonologically unrelated condition, negative or positive mood both elicited a smaller negative waveform than neutral at ROIs of left-anterior (negative mood vs. neutral: *p* = 0.03; positive mood vs. neutral: *p* = 0.03) and mid-central (negative mood vs. neutral: *p* = 0.03; positive mood vs. neutral: *p* = 0.04; Figure 4).

**Figure 5:**
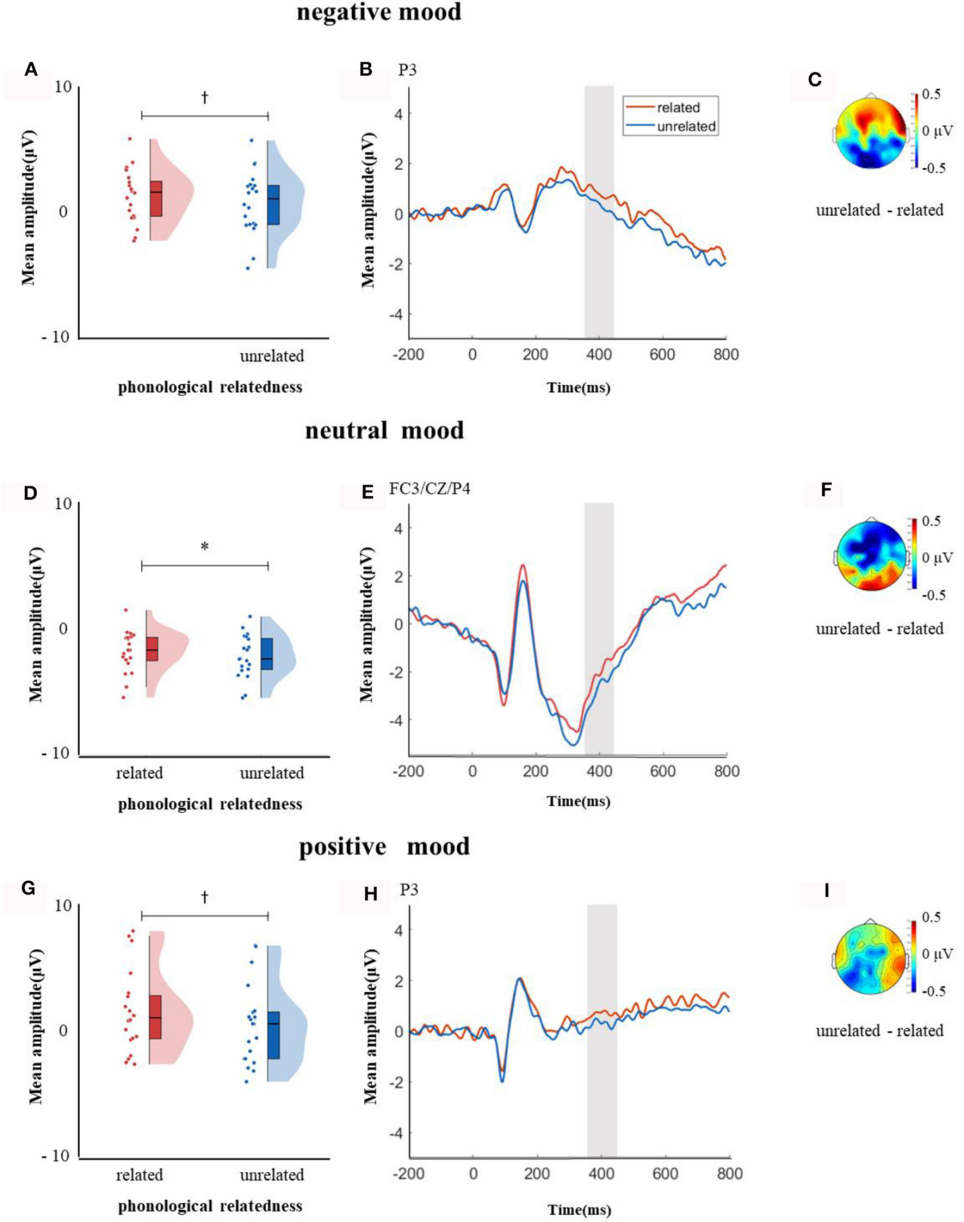
The mean amplitude for young adults in the time window of 350–450 ms (the shadowed interval). **(A, D, G)** Violin plots of phonologically related and unrelated in different induced moods, **(B, E, H)** the grand average waveforms for phonologically related and unrelated in different induced moods, and **(C, F, I)** the map distributions for the significant differences between phonologically related and unrelated in different induced moods, with negativity mood at left-posterior, neutral mood at left-anterior, mid-central and right-posterior, and positive mood at left-posterior. *p < 0.05, ^†^0.05 < *p* < 0.1.

**Figure 6:**
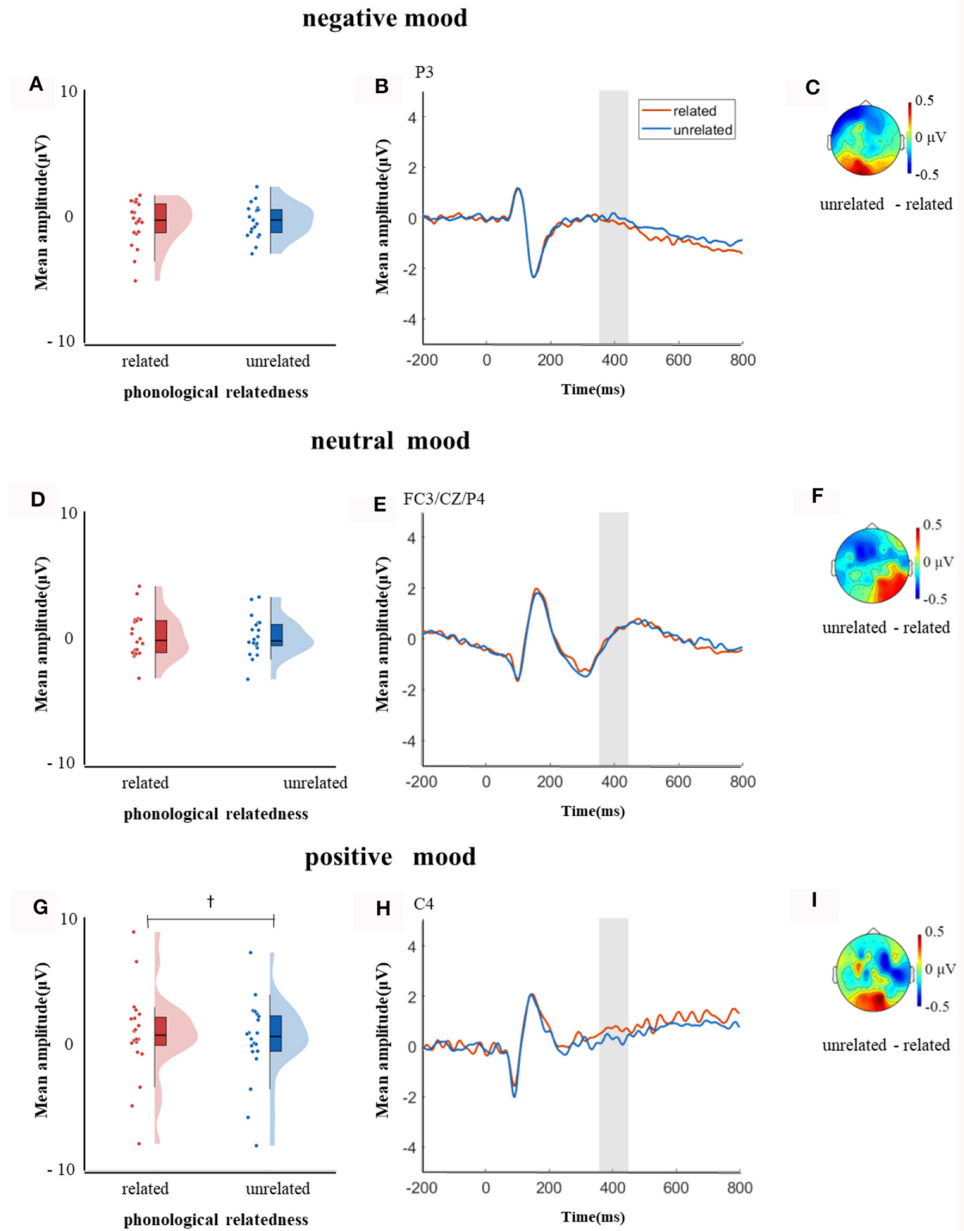
The mean amplitude for older adults in the time window of 350–450 ms (the shadowed interval). **(A, D, G)** Violin plots of phonologically related and unrelated in different induced moods, **(B, E, H)** the grand average waveforms for phonologically related and unrelated in different induced moods, and **(C, F, I)** the map distributions for the significant differences between phonologically related and unrelated in different induced moods, with negativity mood at left-posterior, neutral mood at left-anterior, mid-central and right-posterior, and positive mood at right-central region. ^†^0.05 < *p* < 0.1.

In the time window of 350–450 ms after pictures onset, the effect of age was significant, *F*_(1, 38)_ = 21.09, *p* < 0.001, $\eta_{p}^{2}$ = 0.35, older adults elicited a smaller negative waveform than young adults; and the interaction between age and ROI was significant, *F*_(8, 304)_ = 6.22, *p* < 0.001, $\eta_{p}^{2}$ = 0.14, further simple effect analysis (after *FDR* correction) showed that older adults elicited a smaller negative waveform than young adults at ROIs of left-anterior (*p* < 0.001), left-central (*p* = 0.001), mid-anterior (*p* < 0.001), mid-central (*p* = 0.009), and right-anterior (*p* < 0.001). The triple interaction among phonological relatedness, induced mood, and ROI was significant, *F*_(16, 608)_ = 2.26, *p* = 0.003, $\eta_{p}^{2}$ = 0.06, and further simple effect (after *FDR* correction) analysis showed that in the neutral mood, the phonologically related condition elicited a smaller negative waveform than the unrelated condition at ROIs of left-anterior (*p* = 0.007), mid-anterior (*p* = 0.07), and mid-central (*p* = 0.07). There was no significant difference between phonologically related condition and unrelated condition in both negative and positive moods at all ROIs (all *ps* > 0.1). The quadruple interaction among age, phonological relatedness, induced mood, and ROIs was significant, *F*_(16, 608)_ = 1.78, *p* = 0.03, $\eta_{p}^{2}$ = 0.05, and further simple effect (after *FDR* correction) analysis showed that for young adults in the negative and positive moods, phonologically related condition elicited a lager positive waveform than phonologically unrelated condition at ROIs of left-posterior (negative: *p* = 0.081; positive: *p* = 0.076), while in the neutral mood, phonologically related condition elicited a smaller negative waveform than phonologically unrelated condition at ROIs of left-anterior (*p* = 0.007), mid-central (*p* = 0.049), and right-posterior (*p* = 0.049; Figure 5). While for older adults in the positive mood, phonologically related condition elicited a marginally lager positive waveform than phonologically unrelated condition at ROIs of right-central (*p* = 0.08; Figure 6).

## 4 Discussion

This study investigated the effect of induced moods on phonological encoding in Mandarin Chinese spoken word production for young and older groups. Behavioral results showed that young adults exhibit significant phonological facilitation while older adults do not, suggesting an age-related decline of phonological encoding in spoken word production. Meanwhile, both young and older adults showed an inhibitory effect of negative mood on spoken word production, suggesting older adults preserve the capacity for mood processing. The ERP data revealed distinct patterns of phonological encoding in the two groups. Both groups showed a phonological effect in the time window of 250–350 ms, which reflects phonological encoding. Meanwhile, young adults showed a phonological inhibitory effect around 350–450 ms in negative and positive moods, possibly indicating self-monitoring in speech production. Additionally, induced moods varied in how they modulated phonological effect between older and young adults. Our findings provide initial insight into the interplay of induced mood and linguistic information between young and older groups in Chinese spoken word production.

Behavioral results showed that, compared to neutral and positive moods, individuals in both the young and older groups exhibited longer naming latencies in negative mood, suggesting that different processing styles may be employed in different moods (see also Bodenhausen et al., 1994; Clore and Huntsinger, 2007). Hinojosa et al. (2017) also reported an inhibition effect in spoken production after participants viewed videos that induced negative moods. White et al. (2016) found that in the PWI task, the taboo distractors impeded picture naming compared to neutral and positive distractors. This indicates that negative mood can prompt an analytical and demanding processing style, which requires additional resources to process negative moods and results in an inhibition of naming latencies (see also White et al., 2016; Hinojosa et al., 2017). In contrast, two other studies found that naming latencies were significantly longer in negative or positive images than those in neutral images, and naming latencies were similar in negative and positive images (Hinojosa et al., 2010; Blackett et al., 2017), suggesting that either negative or positive images naming engages similar attentional resources. We used videos to induce emotional moods in the current study, while Hinojosa et al. (2010) and Blackett et al. (2017) used pictures to induce emotional status. Due to the animation of videos, the arousal of motional moods induced by video are higher than those induced by pictures (Gross and Levenson, 1995; Boǧa et al., 2023). Kuperman et al. (2014) proposes an automatic vigilance effect for negative stimuli or threatening stimuli due to their evolutionary importance. Therefore, the vigilance effect induced by videos would be higher than those induced by pictures or images, resulting in longer naming latencies in negative than neutral or positive mood.

Critically, the interaction between induced mood and phonological relatedness was absent in both young and older adults, reflecting that the two factors independently affect naming latencies of spoken word production. The phonological facilitation effect was not affected by different moods. By contrast, White et al. (2016) found an interaction between emotion and phonological relatedness in the PWI task. There is a crucial difference between the two studies: White et al. (2016) manipulated variables of emotion and phonological relatedness on distractor words, whereas in the current study we manipulated moods by watching videos but phonological relatedness on distractor words. We suggest that the induced moods in the current study provide a background that is distinct from the emotion of distractor words provided, and that the induced moods serve as a background to affect spoken word production globally, rather than being limited to a specific stage of spoken production.

One notable finding was that the phonological effect was significant in young but not in older adults, suggesting that spoken word production is subject to a decline in phonological encoding with increasing age. These findings align with previous research indicating that older individuals experience impairments in phonological retrieval (e.g., Oberle and James, 2013) or a reduced phonological facilitation effect compared to young adults (Ouyang et al., 2020). We propose that older adults may not receive adequate activation from phonologically related distractors, which leads to an insignificant phonological effect compared to unrelated distractors. Nevertheless, young and older adults achieved similar performance levels in various induced mood states, indicating the preservation of mood processing in older adults. From a linguistic perspective, our behavioral findings should be interpreted within the theoretical framework of the TDH, which proposes that aging leads to a breakdown in the connection between semantics and phonology in speech.

ERP data revealed a phonological effect around during the 250–350 ms time window in both groups, which falls within the stage of phonological encoding roughly in spoken word production (Indefrey and Levelt, 2004; Indefrey, 2011). The amplitude of the waveform associated with the phonologically related words was smaller than that of the unrelated words. This finding is consistent with previous research (Wang et al., 2017; Feng et al., 2019). We found a phonological relatedness effect in the neutral condition (Zhang and Weekes, 2009; Yang and Zhang, 2015), but this phonological effect was absent in the positive or negative mood condition, suggesting a reduced phonological effect at the electrophysiological level when emotional moods involved in spoken word production. We also found that both young and older adults displayed a smaller negative waveform in response to negative mood than to neutral mood in the phonologically related condition at mid-central region, whereas both groups displayed a smaller negative waveform in response to negative or positive mood than to neutral mood in the phonologically unrelated condition at left-anterior and mid-central regions, reflecting that both groups process the former two moods easier than the neutral mood. According to these findings, we suggest that the induced moods affect the stage of phonological encoding within the time frame of 250–350 ms. Both positive and negative moods had comparable effects on phonological information processing. Speakers exhibited greater attentiveness toward target pictures during negative or positive moods in comparison to neutral mood. This finding is in line with Lang et al.'s (1990, 1997) motivation and attentional model, which proposes that positive and negative moods enhance performances by linking attention to survival needs.

In contrast, another hypothesis suggests that positive or negative moods reduce the attention on target processing, and then the capacity for processing targets decreases (Förster et al., 2006). Studies have shown that participants allocate their cognitive resources to regulate their emotions (Schmeichel, 2007; Whitmer and Banich, 2007), or allocate attentional resources to regulate emotions for changing negative mood to positive mood (Mitchell and Phillips, 2007). Similar results in positive and negative moods were not consistent with the assumption of attentional resources allocation.

In the time window of 350–450 ms, young and older adults presented distinctive patterns for the interaction among induced mood, phonological relatedness, and ROIs. Young adults specifically showed a larger positive waveform in the negative or positive mood at the left posterior in the phonologically related condition when compared with the unrelated condition, reflecting that the underlying mechanism during this time interval is distinct from the time interval of 250–350 ms. It is probable that this component is associated with self-monitoring of phonological information in spoken word production. Self-monitoring in speech involves an internal loop and an external loop. The internal loop monitors abstract phonological codes while the external loop monitors self-generated speech output (Levelt et al., 1999). Wheeldon and Levelt (1995) proposed that internal self-monitoring operates on a syllabified phonological representation, and this process occurs ~355 ms after pictures onset when naming a single word (Indefrey and Levelt, 2004; Indefrey, 2011). Our findings within the time window of 350–450 ms are hence consistent with the time frame of internal self-monitoring.

Two EEG studies provide supports for the view of self-monitoring (Hinojosa et al., 2010; Qu et al., 2012). Using a grapheme monitoring task, Hinojosa et al. (2010) found a component around 400 ms after stimuli onset, and they suggest that this component reflects the time course of grapheme monitoring. Our finding was aligned with the time window around 400 ms which is associated with grapheme monitoring in Hinojosa et al. (2010)'s study. Using a phoneme repetition with picture naming task, Qu et al. (2012) found that phoneme repetition condition elicited a larger negative waveform than not repetition condition around 300–400 ms after pictures onset. They hypothesized that in comparison with phoneme not repetition condition, the monitoring system is under high load to prevent speech errors when initial phonemes are same. In addition, the deflection of component in the time window of 350–450 ms was similar to Qu et al.'s finding. Similarly, in comparison with positive mood, the self-monitoring system is under high processing load in negative mood for checking the possible errors in speaking. Due to this potential difference, we found that a phonological ERP difference was evident in negative mood but not in positive mood in young adults. According to these findings, we cautiously suggest that the component around 350–450 ms may associate with self-monitoring of phonological information during speaking. In this time frame, speakers confirm whether the correct segmental and suprasegmental information are retrieved, which manifests as a meta-cognitive process unconsciously. However, it should be noted that the components observed in the current study and other studies (Hinojosa et al., 2010; Qu et al., 2012) have different map distributions, implying that they may associate with different processing mechanisms, which needs to be investigated further.

In contrast, during the time interval of 350–450 ms, older adults showed a slightly less pronounced waveform for the phonologically related condition in comparison with the unrelated condition in the positive mood, solely at the right-central region, suggesting that older adults might monitor phonemes whilst experiencing positive moods. This finding was consistent with the view that older adults possess a greater preference toward positive information (Murphy and Isaacowitz, 2008) or avoid negative information (Kisley et al., 2007), thus providing evidence for the SST (Carstensen et al., 2000; Charles et al., 2003). Older adults exhibit strong emotional regulation abilities to regulate the influence of negative mood (Charles et al., 2003). Meanwhile, older adults showed lower mood intensity than young adults (Charles et al., 2003), potentially leading to insignificant contrasts among induced moods.

In the neutral mood condition, young adults showed a larger waveform in unrelated condition than in the phonologically related condition within the time window of 350–450 ms across most brain regions. This deflection of waveforms aligns with several studies (e.g., Liotti et al., 2000; Thierry and Wu, 2004; Wu and Thierry, 2010; Wang et al., 2017; Zhang and Damian, 2019; but see Dell'Acqua et al., 2010) in which similarly larger waveforms were found in the phonologically incongruent (or phonologically unrelated) compared to the phonologically congruent (or phonologically related) condition. Generally, this pattern is in line with the findings regarding N400, in which unrelated or violated words with contexts result in a more pronounced waveform than related words to context (Chwilla et al., 2011).

To conclude, the current behavioral and ERP results provide consistent evidence for the decline of phonological encoding in spoken word production with age. The elaborate time course of induced mood and phonological relatedness afforded by EEG provides important insights for investigating the interplay between linguistic information and induced moods in speech production.

## Data availability statement

The raw data supporting the conclusions of this article will be made available by the authors, without undue reservation.

## Ethics statement

The studies involving humans were approved by Independent Ethics Committee of the Department of Psychology, Renmin University of China, Beijing. The studies were conducted in accordance with the local legislation and institutional requirements. The participants provided their written informed consent to participate in this study.

## Author contributions

LJ: Data curation, Formal analysis, Visualization, Writing—original draft. RZ: Conceptualization, Methodology, Software, Writing—original draft. QZ: Conceptualization, Funding acquisition, Investigation, Methodology, Resources, Supervision, Validation, Writing—original draft, Writing—review & editing.

## Footnotes

^1^The coding for the categorical variable is as follows: phonological relatedness (related−0, unrelated−1); age (young−0, older−1); induced mood (neutral−001, positive−010, negative−000).
