# Supplementary material for: The influence of induced moods on aging of phonological encoding in spoken word production: an ERP study
Source: Front Hum Neurosci. 2024 Feb 13;18:1330746. doi: 10.3389/fnhum.2024.1330746 (PMC10896962; doi:10.3389/fnhum.2024.1330746)
Supplement: Supplementary file 1 [file Data_Sheet_1.pdf]

Appendix I: The comprehensive results of ERP analysis

Table S1. The ANOVA analysis of age, phonological relatedness, and induced mood in the time windows of 250-350 ms and 350-450 ms

Variables	250-350 ms			350-450 ms		
	F	***P***	η^2_p	***F***	***P***	η^2_p
age(1,38)	6.37	0.02	0.14	21.09	0.00	0.36
phonological relatedness (1,38)	0.97	0.39	0.03	2.89	0.10	0.07
induced mood (2,76)	4.75	0.04	0.11	0.27	0.76	0.007
age*phonological relatedness (1,38)	0.66	0.42	0.02	0.14	0.71	0.004
age*induced mood (2,76)	0.89	0.42	0.02	1.65	0.20	0.04
phonological relatedness*induced mood(2,76)	0.28	0.75	0.01	0.089	0.92	0.002
age*phonological relatedness*induced mood(2,76)	0.13	0.88	0.003	0.16	0.85	0.004
age*ROI（8,608）	4.72	0.00	0.11	6.22	0.00	0.14
age*induced mood*ROI（8,304）	0.71	0.69	0.02	0.76	0.73	0.02
age*phonological relatedness*ROI（8,608）	0.75	0.58	0.02	0.75	0.64	0.02
phonological relatedness*induced mood*ROI（16,608）	2.40	0.002	0.06	2.25	0.003	0.06
age*phonological relatedness*induced mood*ROI（16,608）	1.17	0.28	0.03	1.78	0.03	0.05

Table S2. The simple effects analysis results of interaction between induced mood, phonological relatedness and ROI in the time windows of 250-350 ms and 350-450 ms

		250-350 ms					350-450 ms				
Variables		Phonologically related		Unrelated		*P*	Phonologically related		Unrelated		*P*
Induced mood	ROI	*M*	*SD*	*M*	*SD*		*M*	*SD*	*M*	*SD*	
Negative mood	Left-anterior	-2.179	0.464	-2.136	0.414	0.852	-0.327	0.434	-0.277	0.38	0.837
	Left-central	-1.174	0.409	-1.109	0.365	0.767	0.531	0.380	0.646	0.337	0.586
	Left-posterior	0.708	0.291	0.538	0.301	0.315	0.318	0.266	0.181	0.275	0.426
	Mid-anterior	-3.133	0.615	-3.169	0.556	0.892	-0.881	0.564	-0.898	0.487	0.955
	Mid-central	-1.828	0.515	-2.061	0.469	0.200	-0.019	0.410	-0.228	0.336	0.309
	Mid-posterior	0.081	0.323	-0.201	0.304	0.127	0.483	0.412	0.251	0.391	0.181
	Right-anterior	-1.936	0.544	-2.109	0.472	0.418	-0.284	0.459	-0.405	0.359	0.640
	Right-central	-0.103	0.335	-0.177	0.320	0.617	0.384	0.373	0.254	0.344	0.456
	Right-posterior	0.891	0.361	0.851	0.397	0.773	-0.359	0.367	-0.439	0.374	0.514
Positive mood	Left-anterior	-2.073	0.620	-1.959	0.664	0.521	-0.437	0.488	-0.385	0.492	0.754
	Left-central	-1.24	0.511	-1.468	0.561	0.213	0.211	0.366	-0.007	0.399	0.176
	Left-posterior	1.144	0.380	0.932	0.374	0.100	0.880	0.347	0.741	0.343	0.100
	Mid-anterior	-3.302	0.831	-3.263	0.858	0.884	-1.350	0.627	-1.468	0.659	0.612
	Mid-central	-2.356	0.615	-2.493	0.668	0.491	-0.667	0.470	-0.716	0.506	0.805
	Mid-posterior	0.615	0.452	0.419	0.441	0.119	1.032	0.440	0.873	0.391	0.334
	Right-anterior	-1.725	0.594	-1.881	0.628	0.341	-0.154	0.447	-0.462	0.473	0.100
	Right-central	-0.082	0.402	-0.109	0.399	0.834	0.240	0.422	0.104	0.416	0.359
	Right-posterior	1.061	0.363	1.083	0.381	0.889	-0.422	0.330	-0.331	0.356	0.59
Neutral mood	Left-anterior	-2.683	0.508	-3.319	0.520	0.002	-0.652	0.428	-1.292	0.463	0.001
	Left-central	-1.242	0.404	-1.523	0.403	0.107	0.492	0.359	0.216	0.362	0.119
	Left-posterior	0.872	0.294	1.066	0.335	0.127	0.645	0.259	0.712	0.291	0.548
	Mid-anterior	-3.606	0.548	-4.185	0.548	0.019	-1.426	0.492	-1.893	0.466	0.036

Mid-central	-3.24	0.503	-3.672	0.485	0.047	-0.919	0.434	-1.283	0.407	0.068
Mid-posterior	0.628	0.384	0.631	0.361	0.986	1.177	0.378	1.228	0.343	0.733
Right-anterior	-2.21	0.407	-2.447	0.417	0.212	-0.632	0.305	-0.749	0.28	0.57
Right-central	-0.246	0.319	-0.432	0.361	0.179	0.167	0.296	0.183	0.319	0.917
Right-posterior	0.856	0.375	1.220	0.397	0.100	-0.450	0.384	-0.047	0.373	0.010

Table S3. The simple effects analysis results of interaction between age, induced mood, phonological relatedness and ROI in the time window of 350~450 ms

Variables			Phonologically related		Unrelated		*p*
Age	Induced mood	ROI	*M*	*SD*	*M*	*SD*	
Young	Negative mood	Left-anterior	-1.762	0.614	-1.574	0.537	0.586
		Left-central	-0.378	0.538	-0.134	0.477	0.414
		Left-posterior	0.826	0.377	0.357	0.388	0.058
		Mid-anterior	-2.514	0.798	-2.229	0.689	0.494
		Mid-central	-0.768	0.579	-0.907	0.475	0.631
		Mid-posterior	0.844	0.582	0.476	0.554	0.134
		Right-anterior	-1.479	0.649	-1.381	0.508	0.79
		Right-central	0.439	0.528	0.349	0.486	0.717
		Right-posterior	-0.014	0.519	-0.319	0.529	0.086
	Positive mood	Left-anterior	-0.983	0.69	-1.051	0.695	0.771
		Left-central	-0.401	0.517	-0.577	0.564	0.436
		Left-posterior	1.351	0.49	1.122	0.485	0.049
		Mid-anterior	-2.804	0.886	-2.924	0.932	0.714
		Mid-central	-1.403	0.664	-1.613	0.716	0.45
		Mid-posterior	1.039	0.623	0.701	0.553	0.15
		Right-anterior	-1.097	0.632	-1.42	0.669	0.15
		Right-central	-0.188	0.597	-0.074	0.589	0.583
		Right-posterior	-0.535	0.467	-0.698	0.503	0.494
	Neutral mood	Left-anterior	-2.237	0.605	-3.152	0.655	0.001
		Left-central	-0.768	0.508	-1.184	0.511	0.097
		Left-posterior	0.663	0.366	0.909	0.411	0.126
		Mid-anterior	-3.567	0.696	-4.084	0.659	0.098
		Mid-central	-1.943	0.613	-2.606	0.575	0.021

		Mid-posterior	0.546	0.535	0.738	0.485	0.363
		Right-anterior	-1.934	0.432	-1.97	0.396	0.899
		Right-central	-0.079	0.418	-0.186	0.451	0.62
		Right-posterior	-0.417	0.543	0.119	0.527	0.015
Older	Negative mood	Left-anterior	1.109	0.614	1.021	0.537	0.799
		Left-central	1.44	0.538	1.426	0.477	0.962
		Left-posterior	-0.19	0.377	0.005	0.388	0.421
		Mid-anterior	0.751	0.798	0.434	0.689	0.446
		Mid-central	0.73	0.579	0.451	0.475	0.337
		Mid-posterior	0.121	0.582	0.026	0.554	0.695
		Right-anterior	0.911	0.649	0.571	0.508	0.355
		Right-central	0.33	0.528	0.159	0.486	0.488
		Right-posterior	-0.704	0.519	-0.56	0.529	0.412
	Positive mood	Left-anterior	0.109	0.69	0.282	0.695	0.464
		Left-central	0.823	0.517	0.562	0.564	0.252
		Left-posterior	0.408	0.49	0.359	0.485	0.663
		Mid-anterior	0.104	0.886	-0.012	0.932	0.726
		Mid-central	0.068	0.664	0.182	0.716	0.683
		Mid-posterior	1.026	0.623	1.046	0.553	0.933
		Right-anterior	0.788	0.632	0.495	0.669	0.19
		Right-central	0.668	0.597	0.283	0.589	0.07
		Right-posterior	-0.309	0.467	0.036	0.503	0.153
	Neutral mood	Left-anterior	0.932	0.605	0.567	0.655	0.167
		Left-central	1.751	0.508	1.616	0.511	0.583
		Left-posterior	0.627	0.366	0.516	0.411	0.485
		Mid-anterior	0.716	0.696	0.298	0.659	0.178

Mid-central	0.106	0.613	0.04	0.575	0.810
Mid-posterior	1.809	0.535	1.718	0.485	0.666
Right-anterior	0.669	0.432	0.472	0.396	0.499
Right-central	0.412	0.418	0.551	0.451	0.521
Right-posterior	-0.484	0.543	-0.213	0.527	0.207

Appendix II: The detailed description of videos

Negative mood (sad)

《Roots and Branches》

Duration: 2 ` 26``

Video Overview: Qi Miao, the youngest sister among the four siblings, was given as a daughter to an elderly couple

Start shot: An elderly couple staring at the little girl

End shot: From bottom to top, we see the crying faces of four siblings

《Warm Spring》

Duration: 1 ` 41``

Video Overview: Grandpa Asks Little Flower about the Situation at Home

Start shot: Grandpa asks the girl, "What's your name?"

End shot: Xiaohua finished saying "Grandpa, please", while Grandpa said "Don't cry" and helped Xiaohua wipe her tears

Positive mood (happy)

《Eat Hot Tofu Slowly》

Duration: 1 ` 49``

Video Overview: Liu Xiaohao slept in class, and the teacher asked him to sing, which made the whole class burst into laughter

Start shot: In the classroom, the teacher is playing the piano

End shot: After Yao Yanan finished singing three words, the whole class burst into laughter.

《A Big Potato》

Duration: 2 ` 22``

Video Overview: Da Lao Hei and cadre Wang are arguing at the dinner table

Start shot: Two girls serve dishes on the table, and the men shout, "Hurry up, serve the table."

End shot: Da Lao Hei takes another bite of food, and Officer Wang looks at him with a slanted eye

Neutral mood (at peace**)**

《Repair Computer》

Duration: 2 ` 02``

Video Overview: Finding the Cause of Touch Screen Damage

Start shot: The master flipped over the laptop

End shot: Master takes away the touchpad and prepares to connect the wires

《IDE Interface Repair》

Duration: 1 ` 11``

Video Overview: Teaching methods for repairing IDE interfaces

Starting shot: The teacher said, "So what is its specific method?"

End shot: The teacher said, "So, this problem can be solved."

Appendix Ⅲ. Stimuli used in the experiment

Target picture names			phonologically related distractor word				
Chinese	English	Pinyin	Chinese	English	Pinyin	Stroke	Frequency
叉子	fork	cha1zi5	岔路	turning	cha4lu4	20	434
灯泡	bulb	deng1pao4	等级	grade	deng3ji2	18	21986
信封	envelope	xin4feng1	新闻	news	xin1wen2	22	262534
眼镜	glasses	yan3jing4	焰火	fireworks	yan4huo3	16	1307
衣架	hanger	yi1jia4	异地	remote	yi4di4	12	4600
尺子	ruler	chi3zi5	池水	pool	chi2shui3	10	941
电话	telephone	dian4hua4	点心	dessert	dian3xin1	13	5549
耳朵	ear	er3duo5	儿子	son	er2zi5	5	78537
牙刷	toothbrush	ya2shua1	亚洲	Asia	ya4zhou1	15	51979
钢笔	pen	gang1bi3	港口	port	gang3kou3	15	14700
蜡烛	candle	la4zhu2	喇叭	horn	la3ba1	17	5358
杯子	cup	bei1zi5	贝壳	shell	bei4ke2	11	2353
铅笔	pencil	qian1bi3	前程	future	qian2cheng2	21	3337
钟表	clock	zhong1biao3	众人	everyone	zhong4ren2	8	22231
帽子	hat	mao4zi5	毛笔	brush	mao2bi3	14	1978
水壶	kettle	shui3hu2	睡眠	sleep	shui4mian2	23	10649
菠萝	pineapple	bo1luo2	伯父	uncle	bo2fu4	11	2829
袜子	sock	wa4zi5	瓦片	tile	wa3pian4	8	558
手表	watch	shou3biao3	兽医	veterinarian	shou4yi1	18	2917
抽屉	drawer	chou1ti4	筹码	chip	chou2ma3	21	1660
钥匙	key	yao4shi5	妖怪	monster	yao1guai4	15	1185
鼠标	mouse	shu3biao1	竖琴	harp	shu4qin2	21	892
桌子	table	zhuo1zi5	酌情	discretion	zhuo2qing2	21	1517
酒杯	goblet	jiu3bei1	救星	saviour	jiu4xing1	20	943
火柴	match	huo3chai2	活人	living	huo2ren2	11	2817
裤子	trousers	ku4zi5	苦难	suffering	ku3nan4	18	9313
衬衫	shirt	chen4shan1	臣民	subject	chen2min2	11	4338
箭头	arrow	jian4tou2	监工	foreman	jian1gong1	13	887
椅子	chair	yi3zi5	疑问	question	yi2wen4	20	14676
沙发	sofa	sha1fa1	傻瓜	fool	sha3gua1	18	5538
葡萄	grape	pu2tao4	瀑布	waterfall	pu4bu4	23	5237

风筝	kite	feng1zheng1	缝隙	gap	feng4xi4	25	2472
梯子	ladder	ti1zi5	题目	topic	ti2mu4	20	11380
树叶	leaf	shu4ye4	叔侄	nephews	shu1zhi2	16	447
公鸡	rooster	gong1ji1	贡献	contribution	gong4xian4	20	58798
玉米	corn	yu4mi3	语文	Chinese	yu3wen2	13	7700
蝴蝶	butterfly	hu2die2	护士	nurse	hu4shi4	10	8005
地球	earth	di4qiu2	敌军	enemy	di2jun1	16	10885
镜子	mirror	jing4zi5	警察	police	jing3cha2	33	40518
冰箱	refrigerator	bing1xiang1	病情	illness	bing4qing2	21	10817
领带	tie	ling3dai4	灵芝	ganoderma	ling2zhi1	13	1957
算盘	abacus	suan4pan2	酸奶	yoghurt	suan1nai3	19	1256
靴子	boot	xue1zi5	穴位	acupoint	xue2wei4	12	2008
手套	mitten	shou3tao4	收入	income	shou1ru4	8	132784
兔子	rabbit	tu3zi5	图表	chart	tu2biao3	16	4524
凉鞋	sandal	liang2xie2	两岸	Cross-strait	liang3an4	15	25440
蜗牛	snail	wo1niu2	卧室	bedroom	wo4shi4	17	10563
眼睛	eyes	yan3jing1	烟囱	flue	yan1cong1	17	2782
火车	train	huo3che1	祸害	scourge	huo4hai5	21	2043
苹果	apple	ping2guo3	乒乓	ping-pong	ping1pang1	12	11863
太阳	sun	tai4yang2	台阶	step	tai2jie1	11	14376
白菜	cabbage	bai2cai4	败仗	defeat	bai4zhang4	13	1631
乌龟	turtle	wu1gui1	武术	martial art	wu3shu4	13	7368
飞机	airplane	fei1ji1	肥皂	soap	fei2zao4	15	3595
大象	elephant	da4xiang4	搭档	partner	da1dang4	22	2025
香蕉	banana	xiang1jiao1	象棋	chess	xiang4qi2	23	6847
拉链	zipper	la1lian4	腊月	twelfth lunar month	la4yue4	16	1917
锤子	hammer	chui2zi5	炊烟	smoke	chui1yan1	18	843
蜻蜓	dragonfly	qing1ting2	庆典	celebration	qing4dian3	14	5374
萝卜	radish	luo2bo5	洛阳	Luoyang	luo4yang2	15	11377

Note: Frequency is the number of occurrences per million.
